# Mark Hallett: scientist and humanitarian in modern movement disorder neurology^☆^

**DOI:** 10.1016/j.prdoa.2026.100460

**Published:** 2026-06-03

**Authors:** Daniel Truong, Joseph Jankovic

**Affiliations:** a Department of Psychiatry and Neurosciences, UC Riverside, Riverside, CA, USA; b The Truong Neuroscience Institute, Orange Coast Memorial Medical Center, Fountain Valley, CA 92708, USA; c Parkinson's Disease Center and Movement Disorders Clinic, Department of Neurology, Baylor College of Medicine, Houston, TX, USA

## Abstract

Mark Hallett, MD (1943–2025) was a highly influential neurologist in the modern history of movement disorders. Over several decades, his work transformed the field from a predominantly descriptive clinical specialty into a neuroscientific discipline. Through pioneering investigations in neurophysiology, cortical excitability, dystonia, tremors, myoclonus, functional movement disorders, and transcranial magnetic stimulation, Hallett helped establish conceptual frameworks that continue to shape contemporary research and clinical practice. Beyond his scientific contributions, he is recognized as a global mentor and educational ambassador whose influence extended across generations of neurologists and neuroscientists worldwide. This article examines Hallett's dual legacy as both a transformative scientist and an academic humanitarian whose career exemplifies the integration of scientific rigor, international collaboration, and humanistic mentorship.

## 1 Introduction

The development of modern movement disorders as a field of neurology during the late twentieth and early twenty-first centuries was driven by a small group of clinician-scientists who fundamentally reshaped the field's intellectual architecture. Among these figures, Mark Hallett, MD occupies a singular position because of his ability to bridge bedside clinical observation with advanced human neurophysiology.

While earlier generations of neurologists focused primarily on phenomenology and anatomical localization, Hallett contributed to a transition toward the understanding of the physiology of movement disorders. His investigations into motor control, cortical inhibition, sensorimotor integration, and maladaptive plasticity provided biologically grounded explanations for disorders that had long remained clinically recognizable but physiologically obscure. In doing so, he helped establish movement disorders as a quantitatively studied branch of systems neuroscience [1], [2], [3], [4], [5].

At the same time, Hallett's influence extended well beyond laboratory discovery. Through mentorship, education, and international collaboration, he became one of the most important academic diplomats in neurology, helping disseminate advanced movement-disorders knowledge across diverse global medical communities [6], [7], [8], [9], [10].

## 2 Scientific contributions

### 2.1 Human neurophysiology and motor control

Hallett's scientific identity is deeply connected to the study of human motor physiology. Working extensively within the National Institutes of Health and the intramural program of the National Institute of Neurological Disorders and Stroke, he contributed to foundational work examining the organization of voluntary movement and cortical motor regulation.

His studies clarified the importance of inhibitory and excitatory networks in motor control, particularly in relation to dystonia and other hyperkinetic disorders. These investigations helped move the field away from simplistic basal ganglia models toward broader network-based understandings of movement dysfunction.

The concept of impaired inhibition in dystonia, now central to modern pathophysiological models, was strongly shaped by work associated with Hallett and his collaborators. Similarly, his contributions to the understanding of maladaptive cortical plasticity influenced subsequent generations of research into movement disorders rehabilitation and neuromodulation [1], [2], [3].

### 2.2 Transcranial magnetic stimulation

Hallett also became one of the leading authorities on transcranial magnetic stimulation (TMS), helping establish it as both an investigative and therapeutic neurological tool [4], [5]. His work contributed significantly to the standardization of TMS methodology and interpretation [4, 11].

Through TMS studies, Dr. Hallett and colleagues advanced understanding of cortical excitability, intracortical inhibition, sensorimotor integration, and neuroplasticity in both healthy individuals and neurological patients [3], [4], [5].

These studies had implications extending beyond movement disorders into stroke, epilepsy, neurorehabilitation, and psychiatric neuromodulation.

### 2.3 Dystonia, tremor, myoclonus, and functional movement disorders

Hallett's contributions to dystonia research are particularly influential. He helped frame dystonia not merely as a disorder of muscle contraction but as a system-level disorder involving abnormalities in sensory processing, cortical plasticity, and motor network regulation.

His work on tremor and myoclonus similarly advanced electrophysiological characterization and classification strategies that improved both diagnosis and mechanistic understanding. Many current neurophysiological approaches used in movement disorders laboratories worldwide can trace conceptual lineage to methodologies developed or refined through his research program.

Using functional MRI, PET and other imaging and neurophysiologic techniques Hallett provided evidence that in functional (previously referred to as psychogenic) movement disorders there appears to be decreased activity of cortical areas implicated in motor preparation, motor selection and feedforward monitoring of movements but increased activity of areas implicated in self-awareness, interoception and emotional processing, coupled with abnormally increased connectivity between limbic and motor regions [5], [6].

### 2.4 Mentorship and global academic influence

Although Hallett's scientific achievements alone would secure his historical prominence, his influence as a mentor may ultimately prove equally enduring.

Over several decades, he trained and collaborated with physicians and scientists from numerous countries across Asia, Europe, Latin America, the Middle East, and North America. Many former trainees subsequently became leaders of movement disorders programs, academic departments, and international neurological societies [6], [7], [8], [9], [10].

In movement disorders neurology, academic lineage carries considerable significance. Training under Hallett became associated not only with technical neurophysiological expertise but also with a model of rigorous, collaborative scientific inquiry. His mentorship style emphasized intellectual openness, interdisciplinary dialogue, and methodological precision.

Importantly, Hallett demonstrated a consistent willingness to support investigators from regions with limited research infrastructure and support. Such efforts contributed to the globalization of movement disorders science during a period when advanced neurophysiological resources were concentrated in relatively few institutions.

### 2.5 Humanitarianism through academic medicine

The humanitarian dimension of Hallett's legacy differs from traditional public health or humanitarian mission models. Rather than focusing primarily on field deployment or disaster medicine, his humanitarianism operated through education, mentorship, and scientific inclusion.

This form of “academic humanitarianism” is often less publicly visible but may possess extraordinary long-term impact. By training international physicians, sharing scientific infrastructure, and fostering cross-border collaboration, Hallett helped build neurological capacity that persisted far beyond individual lectures or research projects.

His leadership role within organizations such as the International Parkinson's and Movement Disorder Society, the International Federation of Clinical Neurophysiology, the American Association of Neuromuscular and Electrodiagnostic Medicine, The International Neurotoxin Association, the Functional Neurological Disorder Society, and the International Association for Parkinsonism and Related Disorders further amplified his influence. Through international congresses, workshops, educational initiatives, and collaborative networks, he contributed to the democratization of advanced movement disorders knowledge worldwide. Mark was known to never turning down an invitation to lecture even in far flung corner of the world sometimes even in the simplest accommodation such as in Mongolia and Vietnam.

### 2.6 Historical position in neurology

Historically, Hallett belongs to the generation of neurologists who transformed movement disorders into a mature neuroscientific discipline. Alongside figures such as Stanley Fahn, Anthony Lang, Joseph Jankovic, David Marsden, and Donald Calne, he contributed to establishing the intellectual foundations upon which current movement disorders research continues to build.

However, Hallett's distinctive legacy lies in the integration of three domains rarely unified at such a high level: major scientific innovation, global educational mentorship, and sustained humanistic professionalism. This combination elevated him beyond the role of a highly productive investigator into that of a field-defining academic statesman.

## 3 Conclusion

Mark Hallett represents a model of the physician-scientist whose influence extends simultaneously through discovery, mentorship, and institutional culture. His work fundamentally advanced understanding of human motor physiology and movement disorders while helping globalize the intellectual infrastructure of the field.

His scientific contributions remain deeply embedded within contemporary neurology, particularly in the neurophysiology of dystonia, cortical plasticity, and motor control. Yet his broader legacy may reside equally in the generations of neurologists he trained and inspired throughout the world.

In an era increasingly defined by technological acceleration and specialization, Hallett's career illustrates the enduring importance of combining scientific excellence with educational generosity and humanitarian academic engagement.

He will be missed not only by his family and friends but by countless collaborators, mentees and patients he touched with his intellect, wisdom, humor and humanity.

We are grateful to all the authors for their contributions to this special issue honoring Mark Hallett.

## CRediT authorship contribution statement

**Daniel Truong:** Conceptualization, Writing – original draft, Writing – review & editing. **Joseph Jankovic:** Conceptualization, Writing – original draft, Writing – review & editing.

## Declaration of competing interest

The authors declare that they have no known competing financial interests or personal relationships that could have appeared to influence the work reported in this paper.
